# *Acinetobacter baumannii* Coordinates Urea Metabolism with Metal Import To Resist Host-Mediated Metal Limitation

**DOI:** 10.1128/mBio.01475-16

**Published:** 2016-09-27

**Authors:** Lillian J. Juttukonda, Walter J. Chazin, Eric P. Skaar

**Affiliations:** a Department of Pathology, Microbiology and Immunology, Vanderbilt University School of Medicine, Nashville, Tennessee, USA; b Departments of Biochemistry and Chemistry and Center for Structural Biology, Vanderbilt University, Nashville, Tennessee, USA; c Tennessee Valley Healthcare Systems, U.S. Department of Veterans Affairs, Nashville, Tennessee, USA

## Abstract

During infection, bacterial pathogens must adapt to a nutrient metal-limited environment that is imposed by the host. The innate immune protein calprotectin inhibits bacterial growth *in vitro* by chelating the divalent metal ions zinc (Zn^2+^, Zn) and manganese (Mn^2+^, Mn), but pathogenic bacteria are able to cause disease in the presence of this antimicrobial protein *in vivo.* One such pathogen is *Acinetobacter baumannii*, a Gram-negative bacterium that causes pneumonia and bloodstream infections that can be complicated by resistance to multiple antibiotics. *A. baumannii* inhibition by calprotectin is dependent on calprotectin Mn binding, but the mechanisms employed by *A. baumannii* to overcome Mn limitation have not been identified. This work demonstrates that *A. baumannii* coordinates transcription of an NRAMP family Mn transporter and a urea carboxylase to resist the antimicrobial activities of calprotectin. This NRAMP family transporter facilitates Mn accumulation and growth of *A. baumannii* in the presence of calprotectin. *A. baumannii* is found to utilize urea as a sole nitrogen source, and urea utilization requires the urea carboxylase encoded in an operon with the NRAMP family transporter. Moreover, urea carboxylase activity is essential for calprotectin resistance in *A. baumannii*. Finally, evidence is provided that this system combats calprotectin *in vivo*, as deletion of the transporter impairs *A. baumannii* fitness in a mouse model of pneumonia, and this fitness defect is modulated by the presence of calprotectin. These findings reveal that *A. baumannii* has evolved mechanisms to subvert host-mediated metal sequestration and they uncover a connection between metal starvation and metabolic stress.

## IMPORTANCE

*Acinetobacter baumannii* is a bacterium that causes bloodstream, wound, urinary tract, and pneumonia infections, with a high disease burden in intensive care units. Treatment of *A. baumannii* infection is complicated by resistance to most antibiotics in use today, and resistance to last-resort therapies has become commonplace. New treatments for *A. baumannii* infection are desperately needed, but our current understanding of the bacterial factors required to cause infection is limited. We previously found that the abundant innate immune protein calprotectin inhibits the growth of *A. baumannii* by withholding essential metals*.* Despite this, *A. baumannii* is still able to infect wild-type mice, which produce calprotectin during infection. Here, we identify factors employed by *A. baumannii* during infection to overcome calprotectin-mediated metal sequestration. Moreover, we expose a connection between metal starvation and metabolism that may be a “chink in the armor” of *A. baumannii* and lead to new treatment options.

## INTRODUCTION

*Acinetobacter baumannii* is a Gram-negative bacterium and an opportunistic pathogen that has emerged as an important cause of infection, especially in critically ill patients (1, 2). While *A. baumannii* is capable of infecting many organs in the human body, this bacterium is most commonly associated with infections of the lung, accounting for 7% of ventilator-associated pneumonias (3). Most *A. baumannii* infections are caused by strains that are resistant to at least three classes of antibiotics, rendering treatment of *A. baumannii* infections challenging (4). Due to the threat posed by *A. baumannii* to human health, it is of paramount importance to improve the current understanding of *A. baumannii* pathogenesis, particularly of the bacterial response to antimicrobial strategies of the host.

A key aspect of host defense against bacterial infection is termed “nutritional immunity,” which includes sequestration of nutrient metals (5). Host-mediated bacterial metal starvation was first described for iron (Fe), but it has now been extended to zinc (Zn^2+^, Zn) and manganese (Mn^2+^, Mn) following the discovery of the multiple metal-chelating properties of the S100 protein calprotectin (6). Calprotectin is a heterodimer of S100A8 and S100A9 (also known as calgranulin A and B, or MRP8 and MRP14) that binds Zn, Mn, and Fe^2+^ ions *in vitro* with high affinity at the dimer interface (7–9).

Calprotectin is important for host defense against *A. baumannii* in the lung. Calprotectin comprises 45% of the cytoplasmic protein in neutrophils (10), and *A. baumannii* infection of the murine lung leads to robust recruitment of neutrophils, which are necessary for bacterial clearance (11). Neutrophil recruitment causes a dramatic accumulation of calprotectin that colocalizes with sites of lobar inflammation and *A. baumannii* colonization (12). Calprotectin-deficient mice have increased bacterial burdens and mortality from *A. baumannii* pneumonia (13). Finally, recombinant calprotectin inhibits *A. baumannii* growth *in vitro*, and this is dependent on an intact hexahistidine Mn binding site within calprotectin; this finding suggests that *A. baumannii* requires Mn for full fitness (8, 13).

Mn is an essential cofactor for life and is predominantly utilized as a redox-active cofactor for enzymes, including superoxide dismutase and ribonucleotide reductase (14). Several families of Mn transporters have been identified in bacteria. The most widely conserved of these are Mn ATP binding cassette (ABC) transporters and the natural resistance-associated macrophage protein (NRAMP) family of Mn transporters, which are important for the virulence of many bacterial pathogens (14). NRAMP family transporters are transmembrane proteins that utilize the proton motive force as an energy source for transport (14). For the pathogen *Staphylococcus aureus*, both an NRAMP family transporter and an ABC family Mn transporter are important for bacterial resistance to calprotectin (15). To date, no Mn transporters have been characterized in *A. baumannii.*

Calprotectin-mediated Mn deprivation restricts *A. baumannii* growth, presumably because Mn-dependent bacterial processes are rendered inactive without their cognate cofactor. However, exactly which bacterial processes are inhibited and how the bacterium responds to these alterations in physiology remain unknown. For instance, multiple metabolic enzymes involved in carbon metabolism, including phosphoglyceromutase (16) and pyruvate carboxylase (17), require Mn or are activated by Mn, but whether central metabolic processes are altered by calprotectin-mediated Mn sequestration is unclear. We hypothesized that understanding the effects of calprotectin exposure on *A. baumannii* physiology *in vitro* may uncover bacterial processes essential for infection in niches where calprotectin is abundant.

The overall goal of this study was to identify mechanisms by which *A. baumannii* overcomes calprotectin-based nutritional immunity. An operon that contains a putative Mn transporter and urea catabolism enzymes from the urea amidolyase family was identified. Based on transcriptional regulation, we hypothesized that urea amidolyase is a component of the *A. baumannii* response to calprotectin-mediated Mn sequestration. Mn transport was demonstrated to be important for growth in the presence of calprotectin and colonization of the murine lung. Urea catabolism was found to be vital for growth in the presence of calprotectin and functionally linked to Mn acquisition in *A. baumannii*. Taken together, these results uncover that host-mediated metal sequestration restricts metabolism in bacterial pathogens, and they broaden the understanding of the bacterial factors required to survive this restriction.

## RESULTS

### *A. baumannii* encodes an NRAMP family transporter that mediates resistance to calprotectin.

**FIG 1:**
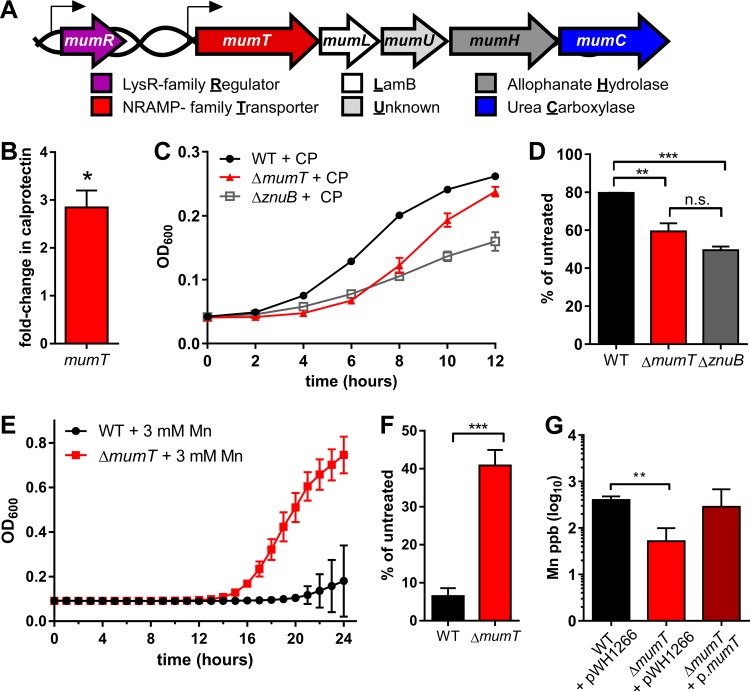
*Acinetobacter baumannii* encodes an NRAMP family Mn importer that mediates resistance to calprotectin. (A) Schematic of the *mum* locus, with gene names and predicted protein functions. (B) *mumT* transcription determined via qRT-PCR. cDNA was reverse transcribed from RNA harvested from wild-type *A. baumannii* in the presence of 125 µg/ml calprotectin. Transcription is graphed as the fold change relative to that in untreated cells. Data were combined from three independent experiments, each performed in technical triplicate, with the means and standard errors of the means graphed. Statistical significance was determined with Student’s *t* test using a reference value of 1.0. (C) Growth of wild-type *A. baumannii* (WT) and the Δ*mumT* and Δ*znuB* mutants treated with 125 µg/ml calprotectin (CP). (D) Growth of *A. baumannii* WT and Δ*mumT* and Δ*znuB* mutant strains treated with 125 µg/ml calprotectin, relative to growth of untreated cells at 8 h. For panels C and D, data were combined from three independent experiments, each performed in technical triplicate, with the means and standard errors of the means graphed. Significance was calculated using a one-way analysis of variance with Tukey’s multiple comparisons test. (E) Growth of *A. baumannii* and Δ*mumT* in LB supplemented with 3 mM MnCl_2_ over time. (F) Growth of *A. baumannii* and Δ*mumT* in 3 mM MnCl_2_ relative to LB alone at 20 h. For E and (F) data are combined from a single experiment performed with three biological replicates with the mean and standard deviation graphed. Data are representative of multiple independent experiments. Significance was calculated using a Student’s *t* test. (G) Mn levels of WT *A. baumannii* with empty vector, strain Δ*mumT* with empty vector, and strain Δ*mumT* with a vector containing *mumT* under control of the 16S promoter grown to mid-log phase. Cells were digested in metal-free nitric acid and analyzed by inductively coupled plasma mass spectrometry. Data are combined from three biological replicates that were measured in technical triplicates, with the means and standard deviations graphed. Significance was calculated using a Student’s *t* test. For all panels, statistical significance is indicated as follows: *, *P* < 0.05; **, *P* < 0.01; ***, *P* < 0.001; n.s., not significant.

We hypothesized that *A. baumannii* is able to overcome calprotectin-mediated Mn chelation by utilizing a metal transporter system that has high affinity for Mn. To identify predicted Mn transporters in *A. baumannii*, the KEGG database (18, 19) was searched for Mn transporter orthologs in the *A. baumannii* ATCC 17978 genome. This search identified only one gene encoding a protein with similarity to NRAMP or ABC family Mn transporters, the gene *A1S_1266. A1S_1266* encodes a potential NRAMP family member. NRAMP family members are integral membrane proteins that transport divalent cations, often having specificity for Mn(II) (14). *A1S_1266* is in a predicted operon containing genes that catabolize urea to ammonia, which we named the manganese and urea metabolism (*mum*) operon (Fig. 1A). As *A1S_1266* is predicted to encode a transporter, this gene was named *mumT* (Fig. 1A).

We hypothesized that *mumT* encodes an Mn importer that is important for growth under Mn-restricted conditions, such as upon exposure to calprotectin. Consistent with this, normalized *mumT* transcript abundance increased upon calprotectin treatment (Fig. 1B)*.* Genetic deletion of *mumT* delayed *A. baumannii* growth in the presence of recombinant calprotectin, as did deletion of the Zn import gene *znuB* (Fig. 1C and D). This growth lag could be complemented by the expression of *mumT* from a plasmid or by the addition of excess Mn (see Fig. S1A to C in the supplemental material). Importantly, growth inhibition of a Δ*znuB* mutant in the presence of calprotectin was only fully rescued by the addition of excess Zn and was not rescued by excess Mn alone (20). We hypothesized that if MumT preferentially imports Mn, loss of *mumT* would increase resistance to toxic levels of Mn but not other divalent cations. In support of this, the Δ*mumT* mutant was able to grow in 3 mM Mn, which is highly toxic to wild-type *A. baumannii* (Fig. 1E and F), whereas the Δ*mumT* mutant was more sensitive than wild-type *A. baumannii* to Fe toxicity and had similar sensitivity to Zn toxicity (see Fig. S1D and E). Finally, to determine whether *mumT* is required for Mn acquisition, cellular Mn concentrations were measured by inductively coupled plasma mass spectrometry (ICP-MS) (Fig. 1G). The Δ*mumT* strain had lower Mn levels in cell pellets than did wild-type *A. baumannii*, and this defect was complemented by expression of *mumT* in *trans*. In contrast, cellular Zn and Fe levels in the Δ*mumT* mutant were not significantly different than the wild-type *A. baumannii* levels (see Fig. S1F). Together, these results demonstrate that *A. baumannii mumT* is important for growth under Mn-restricted conditions and for accumulation of cellular Mn.

### *mumT* upregulation in the presence of calprotectin requires the LysR family transcriptional regulator MumR.

**FIG 2:**
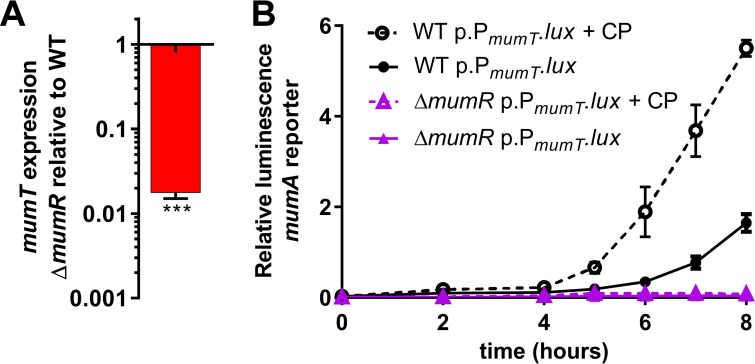
*mumT* is upregulated in the presence of calprotectin via *mumR*. (A) *mumT* transcription in Δ*mumR* mutant cells, measured by qRT-PCR. cDNA was reverse transcribed from RNA harvested from cells grown in LB. Transcription is graphed as the fold change relative to wild-type (WT) *A. baumannii*. Data are combined from three biological replicates that were analyzed in technical triplicate. Statistical significance was determined by using Student’s *t* test with a reference value of 1.0. ***, *P* < 0.001. (B) The *mumT* promoter was cloned in front of the luciferase operon *luxABCDE*. The *mumT* promoter reporter construct was transformed into wild-type *A. baumannii* or strain Δ*mumR.* Luminescence was recorded in cells treated with 125 µg/ml calprotectin (CP) as well as untreated cells. Data are combined from one biological replicate for each strain grown in four technical replicates. The means and standard deviations for results from one representative experiment (of >4) are graphed.

*mumT* is present immediately downstream from a gene that encodes a predicted LysR family transcriptional regulator, *mumR* (Fig. 1A). We hypothesized that *mumR* is required for transcriptional control of *mumT*. To understand the contributions of *mumR* to *mumT* regulation, a strain containing an in-frame deletion of *mumR* was generated. The abundance of *mumT* transcript was significantly decreased in the Δ*mumR* strain relative to that with wild-type *A. baumannii* (Fig. 2A). This suggested that MumR activates *mumT* expression. To confirm this finding, activity of the *mumT* promoter was investigated using a reporter system. The *mumT* promoter was cloned into a plasmid harboring the *Photorhabdus luminescens* luciferase operon, *luxABCDE*, such that activity of the *mumT* promoter resulted in luminescence. This vector was transformed into wild-type *A. baumannii* and Δ*mumR* strain cells, and luminescence was measured under various growth conditions. In rich medium, the *mumT* promoter was active in wild-type *A. baumannii* but not in the Δ*mumR* strain (Fig. 2B)*.* When incubated with 125 µg/ml calprotectin, the activity of the *mumT* promoter was significantly enhanced in wild-type *A. baumannii*, and this was dependent on *mumR.* This result demonstrated that calprotectin exposure induces expression from the *mumT* promoter. Furthermore, it showed that *mumR* is required for upregulation of *mumT* in the presence of calprotectin.

### *mumT* is in an operon with *mumC*, which encodes a urea carboxylase that contributes to *A. baumannii* urea utilization.

Based on the small intergenic distances between the open reading frames (ORFs) for *mumT* (*A1S_1266*) and *mumC* (*A1S_1270*), we predicted that these genes constitute an operon. This was confirmed by performing PCR on cDNA prepared using RNA isolated from wild-type *A. baumannii* (see Fig. S2A in the supplemental material)*.* PCR products were amplified across adjacent ORFs for *mumT*, *mumL*, *mumU*, *mumH*, and *mumC*, and no products were amplified for primers designed to amplify the *mumR-mumT* or *mumC*-*A1S*_*1271* junctions, demonstrating that *mumTLUHC* form an operon.

**FIG 3:**
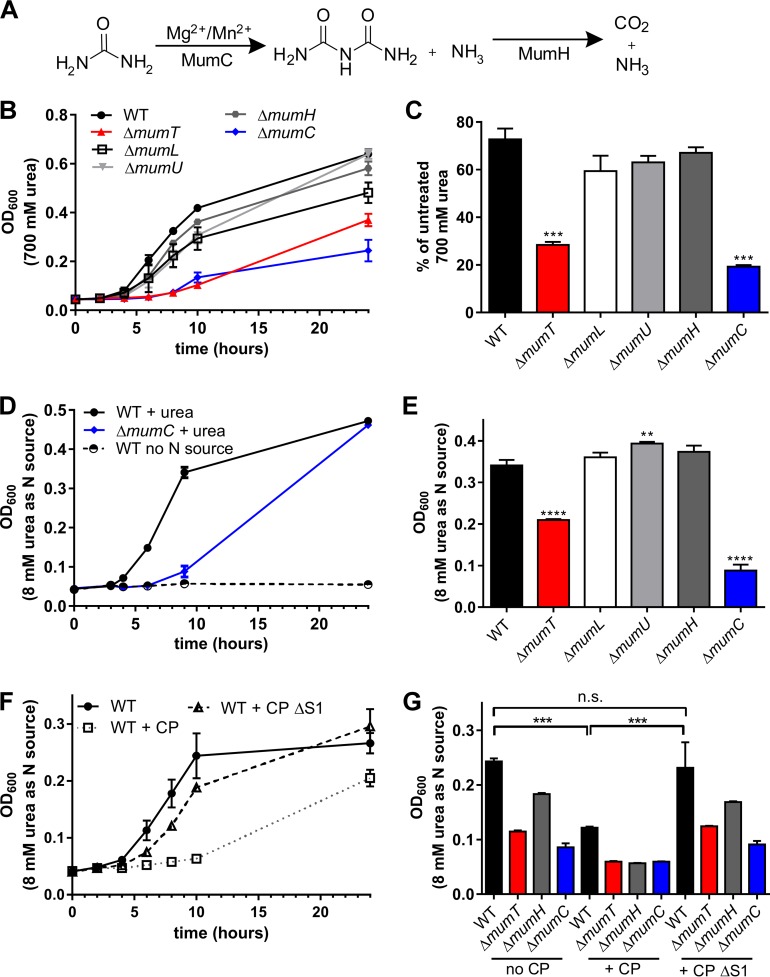
The *mum* operon contributes to *A. baumannii* urea utilization. (A) Predicted reactions catalyzed by urea carboxylase MumC and allophanate hydrolase MumH. Urea is converted to allophanate via biotin-mediated carboxylation of urea by urea carboxylase MumC in a reaction that requires Mg or Mn. Allophanate is hydrolyzed by allophanate hydrolase MumH to carbon dioxide and ammonia. (B) Growth of wild-type *A. baumannii* (WT) and Δ*mumT*, Δ*mumL*, Δ*mumU*, Δ*mumH*, and Δ*mumC* mutant strains in 700 mM urea over time. (C) Growth of wild-type *A. baumannii* and Δ*mumT*, Δ*mumL*, Δ*mumU*, Δ*mumH*, and Δ*mumC* mutant strains in 700 mM urea, relative to growth in LB alone at 24 h. For experiments shown in panels B and C, data are combined from 3 or more independent experiments performed in technical duplicate. Significance was calculated using one-way analysis of variance for each condition, comparing each strain to the wild-type control with Dunnett’s multiple-comparisons test. (D) Growth of wild-type *A. baumannii* and the Δ*mumC* mutant with 8 mM urea as the sole nitrogen source. Wild-type cells grown with no nitrogen source were included as a negative control. (E) Utilization of 8 mM urea as a nitrogen source by wild-type *A. baumannii* and Δ*mumT*, Δ*mumL*, Δ*mumU*, Δ*mumH*, and Δ*mumC* mutant strains. The OD_600_ at 9 h is shown. Data in panels D and E are from a single experiment performed in biological duplicate. Data are representative of 5 independent experiments. Significance was calculated using one-way analysis of variance for each condition, comparing each strain to the wild-type control with Dunnett’s multiple-comparisons test. (F) Wild-type *A. baumannii* growth with urea as primary nitrogen source in the presence of 780 µg/ml calprotectin or calprotectin ΔS1, a variant of calprotectin unable to bind Mn. (G) Growth at 10 h is shown as the raw OD_600_ with urea as the primary nitrogen source with the addition of no calprotectin, 780 µg/ml calprotectin, or 780 µg/ml calprotectin ΔS1, a variant of calprotectin unable to bind Mn. For experiments F and G, data are from a single experiment performed in biological triplicate and are representative of 4 independent experiments. Significance was calculated using one-way analysis of variance for each condition, comparing each strain to the wild-type control with Dunnett’s multiple-comparisons test. ***, *P* < 0.001; ****, *P* < 0.0001.

Next, we sought to identify the functions of members of the *mum* operon. MumH is homologous to allophanate hydrolase, and MumC is a putative member of the biotin carboxylase family. In fungi, the enzyme urea amidolyase contains urea carboxylase and allophanate hydrolase domains. Urea amidolyase converts urea first to allophanate via biotin-mediated carboxylation (urea carboxolyase domain) and then converts allophanate to carbon dioxide and ammonia via hydrolysis (allophanate hydrolase domain) (21). Because *mumH* and *mumC* are adjacent ORFs, we posited that MumC is a biotin-dependent urea carboxylase and MumH and MumC mediate urea degradation (Fig. 3A). Urea amidolyase enzymatic activity has been characterized in enzymes cloned from *Oleomonas sagaranensis* (22, 23), but the roles of urea amidolyase in bacterial physiology and pathogenesis remain unknown.

To evaluate the importance of each *mum* gene in urea degradation, in-frame deletions of *mumL*, *mumU*, *mumH*, and *mumC* were generated. Inactivation of *mumC*, but not of *mumH*, impaired growth in the presence of toxic levels of urea (Fig. 3B and C) but not in LB alone (see Fig. S2B in the supplemental material). While *A. baumannii* cannot grow using urea as a sole carbon source (data not shown), *A. baumannii* can utilize urea as a sole nitrogen source in a manner dependent on the presence of *mumC* (Fig. 3D and E). Importantly, the Δ*mumH* mutant was capable of utilizing urea as a sole nitrogen source (Fig. 3E), which indicated that the single ammonia molecule produced by MumC-mediated urea carboxylation (Fig. 3A) is sufficient to support *A. baumannii* growth as a sole nitrogen source.

Urea carboxylase activity in *Oleomonas sagaranensis* and *Candida utilis* requires either Mg^2+^, Mn^2+^, or Co^2+^ (23, 24), suggesting that *A. baumannii* utilization of urea by MumC may require Mn^2+^. To address this, we chelated Mn by the addition of calprotectin to medium containing urea as the sole nitrogen source. The addition of calprotectin was sufficient to substantially impair growth in urea (Fig. 3F and G). Importantly, growth was not inhibited when a variant of calprotectin (ΔS1) that is unable to bind Mn (8) was added to the medium. Consistent with these findings, *mumT* inactivation decreased growth in high concentrations of urea, which was complemented by the expression of *mumT* in *trans*, demonstrating that the MumT Mn transporter is important to enable urea degradation (see Fig. S2C and D in the supplemental material). Together, these results demonstrated that *mumC* is vital for catabolism of urea in *A. baumannii* and suggest that urea catabolism may be altered by Mn availability.

### *mumC* is important for *A. baumannii* growth in calprotectin.

**FIG 4:**
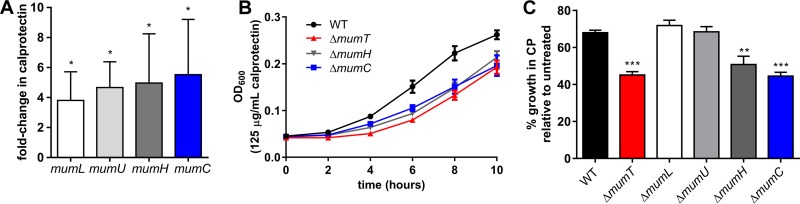
Urea catabolism genes *mumH* and *mumC* are important for growth in calprotectin. (A) *mumT*, *mumL*, *mumU*, *and mumH* transcription following treatment with 250 µg/ml calprotectin. Expression was quantified by qRT-PCR and graphed as the fold change over growth in buffer alone; results were averaged from three independent experiments with each qRT-PCR performed in technical triplicate. Statistical significance was determined with Student’s *t* test using a reference value of 1.0. (B) Growth of wild-type *A. baumannii* (WT) and Δ*mumT*, Δ*mumH*, and Δ*mumC* mutant strains in the presence of 125 µg/ml calprotectin. (C) Percent growth of *A. baumannii* strains in 125 µg/ml calprotectin (CP) relative to growth in buffer alone at 8 h. Growth curves in panels B and C were carried out in rich medium (LB) mixed with calprotectin buffer. Data shown in panels B and C were combined from three independent experiments performed in technical triplicate and depict the means and standard errors of the means. Significance was calculated using a one-way analysis of variance comparing each strain to the wild-type control, with Dunnett’s multiple-comparisons test. *, *P* < 0.05; **, *P* < 0.01; ***, *P* < 0.001.

The entire *mum* operon is upregulated following exposure to calprotectin (Fig. 4A). Based on this observation, we hypothesized that additional *mum* genes may be important for growth in calprotectin. The sensitivities of *A. baumannii* Δ*mumL*, Δ*mumU*, Δ*mumH*, and Δ*mumC* mutant strains to calprotectin growth inhibition were evaluated (Fig. 4B). The strains lacking *mumL* and *mumU* exhibited sensitivity to calprotectin similar to that of wild-type *A. baumannii* (Fig. 4C; see also Fig. S3A in the supplemental material). This demonstrated that *mumL* and *mumU* are not important for growth in the presence of calprotectin. In contrast, the Δ*mumH* and Δ*mumC* mutants, strains harboring in-frame deletions of allophanate hydrolase and urea carboxylase, respectively, had increased sensitivity to calprotectin (Fig. 4B and C). The Δ*mumC* mutant did not exhibit decreased transcription of *mumT* (see Fig. S3B). The growth deficit of the Δ*mumC* mutant in calprotectin could be complemented by the addition of exogenous Mn to the medium (see Fig. S1C in the supplemental material) or by providing a copy of *mumC* in *trans* (see Fig. S3C and D). Importantly, the Δ*mumC* strain did not exhibit resistance to Mn toxicity (see Fig. S3E), suggesting that its role in calprotectin resistance is not related to Mn transport. These data indicate that urea catabolism via *mumH* and *mumC* is important for *A. baumannii* resistance to calprotectin.

### *mumT* contributes to the fitness of *A. baumannii* in a murine pneumonia model.

**FIG 5:**
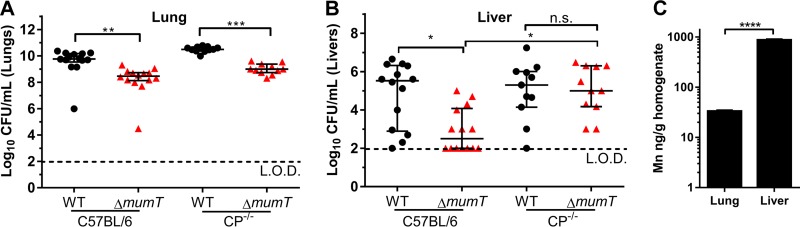
*mumT* contributes to the fitness of *A. baumannii* during murine pneumonia. Mice were intranasally inoculated with a 1:1 mixture of wild-type (WT) *A. baumannii* and strain Δ*mumT*. Following 36 h of infection, mice were euthanized and lungs (primary infection) and livers (dissemination) were harvested, and bacteria were enumerated by dilution plating on nonselective medium and medium containing kanamycin. (A) Bacterial burdens of wild-type and strain Δ*mumT* recovered from lungs of C57BL/6 and CP^−/−^ mice. The mean results and standard deviations are indicated by horizontal lines. Significance was calculated using one-way ANOVA with Tukey’s multiple-comparisons test. (B) Bacterial burdens of wild-type and strain Δ*mumT* recovered from livers of C57BL/6 and CP^−/−^ mice. Median and interquartile range data are indicated by horizontal lines. Significance was calculated using a Kruskal-Wallis test with Dunn’s multiple-comparisons test. For panels A and B, each symbol represents the burden recovered from an individual mouse, and the results of two independent experiments were combined. The limit of detection was 100 CFU per organ and is indicated by the dashed line in both panels. (C) Mn levels in *A. baumannii*-infected livers and lungs were measured by ICP-MS. Organs harvested from three mice were used for this analysis. Significance was calculated using Student’s *t* test. *, *P* < 0.05; **, *P* < 0.01; ***, *P* < 0.001; ****, *P* < 0.0001.

To investigate the contribution of *mumT* to *A. baumannii* fitness in the murine lung, C57BL/6 mice were inoculated intranasally with a 1:1 mixture of wild-type *A. baumannii* and the Δ*mumT* mutant*.* After 36 h, bacterial burdens were quantified from the lungs and the liver, a site of systemic dissemination (Fig. 5A and B). Strain Δ*mumT* burdens were significantly lower than wild-type burdens in both the lungs and the livers of C57BL/6 mice, indicating that *mumT* contributes to fitness in this infection model. To define the role of calprotectin in the fitness defect of the Δ*mumT* strain, calprotectin-deficient mice (S100A9^−/−^) were also coinfected with wild-type *A. baumannii* and the Δ*mumT* strain*.* As in C57BL/6 mice, Δ*mumT* strain burdens were significantly lower than those in the wild type in the lungs of calprotectin-deficient mice. However, the fitness deficit of the Δ*mumT* strain in the liver was completely rescued in the absence of calprotectin. These findings indicate that calprotectin is vital for limiting *A. baumannii* Δ*mumT* strain dissemination to the liver. To elucidate whether the differential rescue of the Δ*mumT* mutant in the liver and the lung of calprotectin-deficient mice correlates with Mn concentrations, *A. baumannii*-infected livers and lungs were subjected to ICP-MS analysis. Mn abundance in the liver was over 25-fold greater than Mn abundance in the lung (Fig. 5C). The high level of Mn in the liver correlated with increased fitness of the Δ*mumT* strain in the liver relative to that in the lung in calprotectin-deficient mice; this indicated that the liver of a calprotectin-deficient mouse is Mn replete. The result indicating that the Δ*mumT* strain is attenuated in the livers of wild-type mice suggests that calprotectin is sufficient to Mn starve *A. baumannii* even in the Mn-abundant liver. Taken as a whole, these data reveal that *mumT* is important for *A. baumannii* fitness during infection and demonstrate that calprotectin is important for preventing Δ*mumT* strain dissemination to the murine liver.

### The *mum* system is broadly conserved across bacteria.

**FIG 6:**
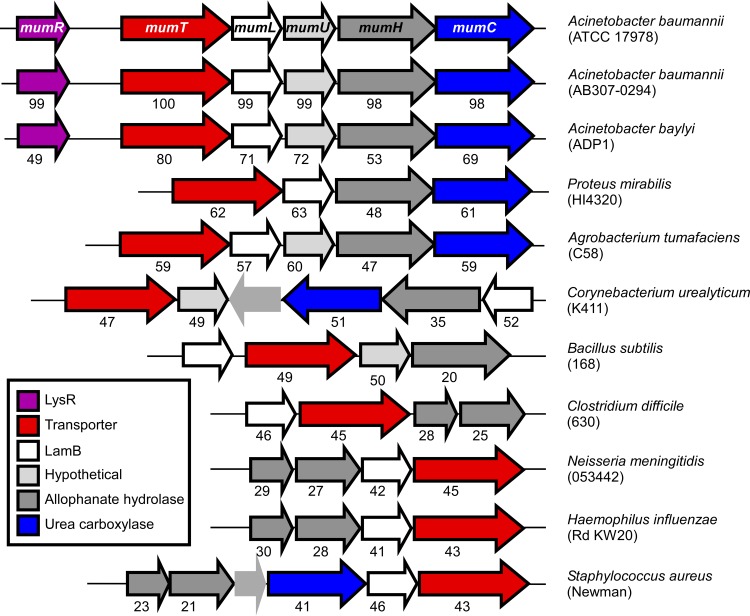
The *mum* system is broadly conserved across bacteria. Genetic alignments of representative organisms that are predicted to contain orthologs of the *mum* system in adjacent loci. Eleven organisms are depicted of the 88 total organisms identified in the SEED database. The numbers underneath each gene correspond to amino acid similarity, based on Clustal W2 alignment to the representative *A. baumannii* genes. Genes shown in light grey with no outline are not part of the *A. baumannii* mum operon.

Calprotectin has antimicrobial activity against numerous pathogens (8). Because of the importance of the *mum* operon in *A. baumannii* resistance to calprotectin, the conservation of this system was investigated (Fig. 6). The *mum* operon was present in all *A. baumannii* strains queried and other *Acinetobacter* species. A *mumR* homolog was present adjacent to the *mum* operon in all *Acinetobacter* species interrogated but was not present outside the *Acinetobacter* genus, suggesting that *Acinetobacter* has evolved with a unique regulatory mechanism for this operon. The *mum* operon is present with at least four of the five genes retained in some other *Gammaproteobacteria*, including the urinary pathogen *Proteus mirabilis*, and more distantly related *Proteobacteria*, including *Agrobacterium tumefaciens*. Portions of the *mum* operon, including NRAMP family transporters and allophanate hydrolase genes, are also present in diverse bacterial phyla, including *Actinobacteria* and *Firmicutes*. Importantly, portions of the *mum* operon are present in diverse bacterial pathogens, including *S. aureus* and *Neisseria meningitidis*. These observations indicate that the *mum* operon is broadly conserved across bacteria but not in other domains of life.

## DISCUSSION

**FIG 7:**
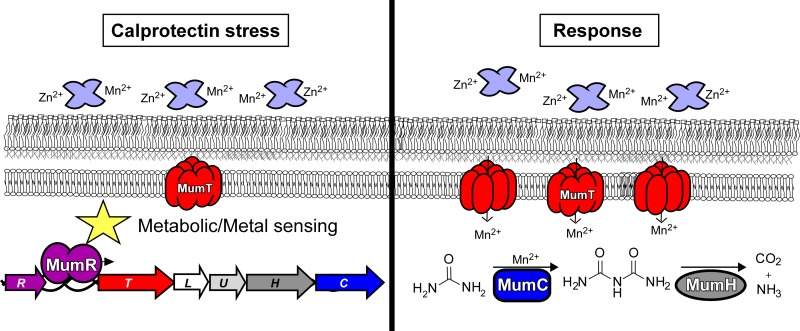
Model for the *mum* system response to calprotectin-mediated manganese sequestration. Calprotectin induces *A. baumannii* Mn starvation and an adaptive response. *mum* activation is orchestrated by the transcriptional regulator, MumR, which enhances *mum* expression. *mumT* encodes an NRAMP family transporter, MumT, that increases cellular Mn content by facilitating Mn import into the cytoplasm. *mumT* increases *A. baumannii* fitness in the lung, presumably by providing Mn for important intracellular processes. *mumC* encodes a urea carboxylase enzyme, MumC, that likely utilizes Mn as cofactor. MumC catabolizes urea to ammonia and carbon dioxide, enables the use of urea as a sole nitrogen source, and provides resistance to calprotectin.

*A. baumannii* colonization of the murine lung generates a robust immune response, which ultimately results in copious amounts of calprotectin being present at the host-pathogen interface (12). Here, we demonstrated that the *mum* operon responds to calprotectin and contributes to *A. baumannii* calprotectin resistance (Fig. 7). MumT was established as a Mn transporter, the unique transcriptional regulation of *mumT* by *mumR* was determined, and a link between *mumT* and urea catabolism via *mumC* was identified. Additionally, urea catabolism was identified as the first metabolic pathway linked to calprotectin resistance, an important step in identifying the mechanisms by which calprotectin disrupts bacterial physiology and inhibits bacterial growth during infection. Finally, the *mum* system was demonstrated to be important for *A. baumannii* fitness in the murine lung and liver, and calprotectin was found to be required by Mn-starved *A. baumannii* in the liver.

*mumT* encodes an NRAMP family homolog. *mumT* is unique compared to previously identified NRAMPs, as MumT shares low sequence homology with reported NRAMPs (<25%), and NRAMPs are typically monocistronic (25). Inactivation of the *S. aureus* NRAMP family member MntH increases sensitivity to calprotectin, although a second Mn transporter (MntABC) must be deleted to see a dramatic growth difference in the presence of calprotectin (15). Similar to this finding, deletion of *mumT* delays *A. baumannii* growth in the presence of calprotectin, and this growth difference is reversed by the addition of excess Mn to the medium. In contrast to results obtained with *S. aureus*, *A. baumannii* growth is significantly decreased by inactivation of *mumT* alone, suggesting that *A. baumannii* may not encode another high-affinity Mn import system. Previously reported NRAMP family transporters have varied specificities for Mn, Fe, and other divalent cations (26–31). The metal specificity of MumT was evaluated by determining sensitivity to toxic levels of Mn, Fe, and Zn. The Δ*mumT* strain was less sensitive than wild-type *A. baumannii* to Mn toxicity. This is consistent with the decreased ability of this strain to accumulate Mn, as measured by ICP-MS. Together, the increased sensitivity of the Δ*mumT* mutant to calprotectin and decreased sensitivity of the Δ*mumT* mutant to toxicity of Mn strongly suggest that *mumT* encodes a transporter that imports Mn. Interestingly, the Δ*mumT* strain had increased sensitivity to Fe toxicity. We hypothesize that the enhanced sensitivity of the Δ*mumT* strain to Fe toxicity stems from disruption of the Mn:Fe ratio. This is consistent with the finding that the Mn:Fe ratio is important for *Neisseria meningitidis* to survive metal toxicity (32).

The Δ*mumT* mutant was less fit than wild-type *A. baumannii* for colonizing the lung and dissemination to the liver. Of note, the Δ*mumT* strain was not attenuated in dissemination to the livers of mice lacking calprotectin, consistent with the model showing that calprotectin is required to starve *A. baumannii* of Mn and prevents colonization of the liver. *S. aureus* inactivated for Mn transporters *mntH* and *mntABC* is also attenuated in the livers of wild-type but not calprotectin-deficient mice (15). Similarly, *A. baumannii* inactivated for the Zn transporter *znuABC* is significantly attenuated in the livers of wild-type mice but not significantly attenuated in calprotectin-deficient mice (13). Together, these findings implicate calprotectin metal sequestration as particularly important in host defense of the liver relative to other organs. The heightened efficacy of calprotectin in the liver may be because the liver, the site of Mn absorption into the systemic circulation and Mn excretion into bile, is the most Mn-replete organ in the body (33). The concentrations of Mn in the *A. baumannii*-infected liver are approximately 25-fold higher than Mn concentrations in the *A. baumannii*-infected lung. In this setting of excess Mn, the Δ*mumT* mutant appeared to be capable of importing sufficient Mn through other transport systems, unless calprotectin was present to sequester Mn. The Δ*mumT* strain was less fit than wild-type *A. baumannii* in the lungs of mice lacking calprotectin, suggesting that additional stresses beyond calprotectin exist in the lung that decrease the fitness of this mutant strain.

Unlike other reported NRAMP family transporters, *mumT* is in an operon. The other genes in this operon were not previously described in *A. baumannii* and lacked an obvious link to Mn homeostasis. A predicted function was not identified for *mumU* or *nml*, but based on sequence homology, *mumH* and *mumC* are predicted to encode allophanate hydrolase and urea carboxylase, enzymes that catalyze the biotin- and ATP-dependent two-step catabolism of urea to ammonia and carbon dioxide. Homologs of these genes in *O. sagaranensis* have been cloned and their enzymatic activities verified (22, 23); however, allophanate hydrolase in *Pseudomonas* functions in cyanuric acid metabolism, not urea metabolism (34). Therefore, the physiological role of these enzymes in bacteria can vary. *A. baumannii* utilized urea as a nitrogen source but not a carbon source, and urea nitrogen utilization depends on *mumC.* These results demonstrate that urea carboxylase has a physiological role in urea catabolism in this bacterium.

Since *mumC* is in an operon with *mumT*, we investigated whether *mumC* requires *mumT-*delivered Mn for activity. Previous reports suggested that urea carboxylase activity requires divalent cations (23, 24). A strain inactivated for *mumT* was impaired for growth in urea as a sole nitrogen source and in the presence of toxic levels of urea. Furthermore, this effect was specific to inactivation of *mumT* and does not occur when other genes in the operon are inactivated. Therefore, this finding suggests that *mumC-*mediated urea catabolism is Mn dependent.

It is well established that calprotectin inhibits bacterial growth *in vitro* and hampers the growth of some bacteria during infection (35). Ostensibly, calprotectin inhibits growth by suppressing metal-dependent bacterial processes. However, it is unclear what specific bacterial processes are inhibited and how this affects bacterial physiology; currently, the only bacterial process known to be inhibited by calprotectin is *S. aureus* superoxide dismutase activity (36). Because evolutionary conservation of genomic organization can suggest similar function, we hypothesized that other genes in the *mum* operon may be important for resistance to calprotectin. In keeping with this, inactivation of *mumC* significantly decreased growth in the presence of calprotectin. This result demonstrated that urea degradation increases the ability of *A. baumannii* to combat calprotectin metal limitation. Furthermore, calprotectin, but not calprotectin lacking the ability to tightly bind Mn, completely inhibited growth of *A. baumannii* utilizing urea as a sole nitrogen source. One interpretation of these results is that urea degradation is a Mn-, Zn-, or Fe-dependent process that is inhibited by calprotectin.

The question remains: why is urea degradation important for growth in the presence of calprotectin? Urea is generated as a by-product of metabolism in rich medium, and calprotectin-mediated metal starvation may cause a metabolic strain by inhibiting metal-dependent metabolic processes. This could lead to a buildup of urea that requires *mumC*-mediated breakdown. Future work to query this hypothesis will also help define metabolic pathways in *A. baumannii*. Alternatively, urea and/or ammonia could serve as a signaling molecule within the bacterial cell. Our findings emphasize the importance of improving the understanding of *A. baumannii* metabolism and the role of metabolism in *A. baumannii* virulence. In this regard, we report that calprotectin-mediated metal starvation and urea catabolism are linked in *A. baumannii.*

The finding that urea carboxylase is important for defense against the antimicrobial protein calprotectin *in vitro* extends the known role of urea in microbial pathogenesis. There are two described pathways for catabolizing urea in bacteria: urease and urea amidolyase (37). Urease is a key virulence factor for *Helicobacter pylori*, as it is required for local alkalinization and chemotaxis in the stomach (38–40). *P. mirabilis* also utilizes urease as a virulence factor in the bladder, the site of host urea excretion; urease activity of *P. mirabilis* alters the pH and causes calculus formation in urine (41). Urease is also required for virulence of the fungal pathogens *Cryptococcus neoformans* (42) and *Coccidioides posadasii* (43). The only reported virulence role for urea amidolyase systems is for *C. albicans*, which uses urea-produced ammonia to regulate pH and induce the yeast-to-hypha transition; this system is important for escape from macrophages and colonization of the kidney (44, 45). The present study indicates that urea amidolyase systems are also important for defense against the human antimicrobial protein calprotectin.

The *mum* operon is conserved across many, but not all, bacteria. The *mum* operon is present in many nonpathogenic organisms, including *Acinetobacter baylyi*, suggesting this operon did not evolve exclusively as a virulence factor. However, it is present in many pathogens, including *S. aureus*. Therefore, a better understanding of the genes within this operon may reveal drug targets for the treatment of multidrug-resistant infections.

## MATERIALS AND METHODS

### Bacterial strains and reagents.

The strains used in this study are described in Table S1 in the supplemental material. All strains are derivatives of the human clinical isolate *A. baumannii* ATCC 17978. Cloning was performed in *Escherichia coli* DH5α. Bacteria were routinely grown in lysogeny broth (LB) at 37°C unless otherwise noted. Solid medium contained 1.5% agar. Antibiotics were added at the following concentrations for *A. baumannii* and *E. coli*, respectively: 500 µg ml^−1^ and 100 µg ml^−1^ ampicillin, 40 µg ml^−1^ kanamycin, 10 µg ml^−1^, and 5 µg ml^−1^ tetracycline. All antibiotics were purchased from Sigma (St. Louis, MO). In-frame deletion strains (Δ*mumR*, Δ*mumT*, Δ*mumL*, Δ*mumU*, Δ*mumH*, and Δ*mumC* mutant strains) were generated via homologous recombination utilizing the suicide plasmid pFLP2 and screened by PCR and Southern blotting as previously described (46). Some constructs were generated by ligating the stitched PCR product directly into pFLP2 (for the Δ*mumR* and Δ*mumT* mutants) or by using Gibson recombineering (for the Δ*mumL*, Δ*mumU*, Δ*mumH*, and Δ*mumC* mutants) (New England Biolabs, Ipswich, MA). Primers used to generate in-frame deletion strains, complementation plasmids, and reporter plasmids are listed in Table S2 in the supplemental material. Complementation vectors for the Δ*mumT* and Δ*mumC* strains were constructed in pWH1266 under control of the 16S promoter (*r01*) as previously described (46), except that complementation vectors did not include a c-Myc tag and the *mumC* complementation vector was cloned between EcoRV and BamHI sites. p.*r01*.*WH1266* was used as the empty-vector control. Antibiotic selection of strains containing the pWH1266 plasmid used 500 µg ml^−1^ ampicillin. Luciferase promoter reporter constructs were generated in pMU368 and derivatives (47). The *Photorhabdus luminescens* luciferase operon *luxABCDE* was PCR amplified from pXen1 (48) with primers that included 5′ BamHI and 3′ SpeI restriction sites; the products were then digested and ligated into pMU368 to generate p.*luxABCDE*.MU368. To permit selection of the pMU368 plasmid in kanamycin-resistant deletion strains, a tetracycline resistance cassette was PCR amplified from AB0057 genomic DNA and ligated into p.*luxABCDE*.MU368 at the KpnI restriction site to create p.*luxABCDE*.MU368.*tet*. The ligation product was transformed into DH5α and selected on 5 µg ml^−1^ tetracycline–LB agar, and the plasmid was purified. An approximately 300-bp segment of the *mumT* promoter was PCR amplified with primers that included 5′ SacI and 3′ BamHI restriction sites, restriction digested, and ligated into p.*lux*.*ABCDE*.MU368.*tet* to generate p.P*_mumT_*.*luxABCDE*.MU368.*tet.* The ligation product was transformed in DH5α, plasmid purified by using a miniprep kit, and transformed into wild-type *A. baumannii* or Δ*mumR* mutant cells*.* Antibiotic selection of strains containing the p.MU368.*tet* plasmid was achieved by using 10 µg ml^−1^ tetracycline. Recombinant human calprotectin was expressed and purified as previously described (35).

### Bacterial growth assays.

Unless otherwise stated, all growth assays were carried out in 96-well flat-bottom plates in 100-µl volumes following inoculation with 1-µl aliquots of overnight cultures. The optical density at 600 nm (OD_600_) was measured as a proxy for growth.

### (i) Urea and metal toxicity assays.

All urea and metal toxicity assays were carried out in LB medium with the addition of freshly prepared, sterile metal or urea stocks. Stock solutions (10 M) of urea were prepared in LB, and the final volume of each well was 90 µl. Stocks containing 100 mM MnCl_2_, 100 mM ZnCl_2_, or 100 mM FeSO_4_ were prepared in water, filter sterilized, and used immediately.

### (ii) Calprotectin antimicrobial growth assays.

Calprotectin antimicrobial growth assays were performed using methods similar to those previously described (8). Briefly, overnight cultures were subcultured 1:50 in LB for 1 h prior to inoculation of a 1:100 dilution containing 40% LB and 60% calprotectin buffer (100 mM NaCl, 3 mM CaCl_2_, 5 mM β-mercaptoethanol, 20 mM Tris; pH 7.5) and titration of calprotectin with or without supplementation of MnCl_2_ at the indicated concentrations.

### (iii) *mumT*-reporter luminescence assay.

For reporter luminescence assays, *A. baumannii* strains harboring luminescence reporter plasmids were grown in medium containing a titration of calprotectin in 60% LB and 40% calprotectin buffer. Cultures were grown in black-sided 96-well plates (Corning), and luminescence was measured using a plate reader (BioTek, Winooski, VT).

### (iv) Sole nitrogen source growth assays.

Sole nitrogen source growth assays were performed in modified E medium lacking nitrogen sources (49) and containing the following: 28 mM K_2_HPO_4_, 28 mM KH_2_PO_4_, 1 mM MgSO_4_, 50 mM acetate (carbon source), and a modified Vishniac’s trace minerals mix (650 µM Na_2_EDTA, 90 µM FeSO_4_, 13 µM MnCl_2_, 8 µM CuCl_2_, 4.5 µM Na_2_MoO_4_, 33.5 µM CoCl_2_, 68 µM ZnCl_2_) (50). Urea was added at the indicated concentrations.

### Mouse infections.

A mouse pneumonia model of *A. baumannii* infection was employed as previously described (13). Briefly, wild-type *A. baumannii* or the Δ*mumT* mutant strain was freshly streaked from frozen stocks onto LB agar or LB agar containing 40 µg ml^−1^ kanamycin, respectively, 2 days prior to infection. Overnight cultures were grown in LB without antibiotic selection. On the day of the infection, overnight cultures were subcultured to 1:1,000 in 10 ml of LB and grown to mid-exponential phase. Cells were then harvested by centrifugation, washed twice in phosphate-buffered saline (PBS), and resuspended in PBS to a final concentration of 1 × 10^10^ CFU ml^−1^. Wild-type and Δ*mumT* strain suspensions were then combined in a 1:1 ratio, mixed thoroughly, and immediately utilized for infection. Mice were anesthetized via intraperitoneal injection of 2,2,2-tribromoethanol diluted in PBS. Anesthetized mice were inoculated intranasally with 5 × 10^8^ CFU in 50-μl volumes. Infections proceeded for 36 h. Mice were then euthanized by use of CO_2_, and lungs and livers were removed and placed on ice. Organs were homogenized in 1 ml PBS, serially diluted in PBS, and dilutions were spot plated onto LB agar and LB agar containing 40 µg ml^−1^ kanamycin. Strain Δ*mumT* burdens were enumerated by counting colonies recovered on kanamycin-containing plates. Infections were performed at the Vanderbilt University Medical Center under the principles and guidelines described in the National Research Council’s *Guide for the Care and Use of Laboratory Animals* (54) and using Institutional Animal Care and Use Committee (IACUC)-approved protocol M/10/165. Vanderbilt University Medical Center is an American Association for Laboratory Animal Science (AALAS)-accredited facility. Vanderbilt University Medical Center is registered with the Office of Laboratory Animal Welfare (OLAW), assurance number A-3227-01.

### Inductively coupled plasma mass spectrometry.

To prepare bacterial samples for ICP-MS analysis, bacterial cultures were grown overnight in LB containing 500 µg ml^−1^ ampicillin and subcultured 1:50 in LB containing 500 µg ml^−1^ ampicillin. Bacteria were subcultured 1:50 for 1 h, and then cultures were diluted 1:100 into 10 ml of 60% LB–40% calprotectin buffer containing 500 µg ml^−1^ ampicillin and grown for 8 h. Bacterial cultures were then transferred to preweighed metal-free 15-ml conical tubes (VWR, Radnor, PA). Pellets were harvested by centrifugation, washed twice with Milli-Q deionized water, and dried. The pellet weight was then recorded using an analytical balance (Mettler-Toledo, Columbus, OH). Pellets were digested with 1 ml 50% HNO_3_ (optima-grade metal-free; Fisher, Waltham, MA) at 50°C overnight, diluted with 9 ml Milli-Q deionized water, weighed using an analytical balance, and subjected to mass spectrometry. Whole organs from *A. baumannii*-infected mice were homogenized in 1 ml PBS and digested in 2 ml HNO_3_ and 500 µl H_2_O_2_ (optima-grade metal-free; Fisher, Waltham, MA) at 90°C overnight in metal-free Teflon jars for digestion. Digested samples were then diluted with 9 ml Milli-Q deionized water and submitted for ICP-MS analysis at the Vanderbilt Mass Spectrometry Research Center. Levels of ^66^Zn, ^55^Mn, and ^56^Fe were measured, concentrations were determined by utilizing a standard curve for each metal, and results were normalized by dilution factor.

### Determining the conservation of the *mum* operon *in silico*.

The “compare region” feature of the SEED viewer (51) was used to identify genomic regions similar to the *mum* operon, with *mumT* set as the focus gene. From the 58 *Archaea*, 962 *Bacteria*, and 562 *Eukarya* genomes in the SEED database at the time of query, 88 were found to include sets of genes with similar sequences. Genomic regions from 11 organisms were selected for protein alignments. Protein sequences were downloaded from the SEED database and aligned by using ClustalW2 (52, 53) for comparison to the 17978 homolog.

### Quantitative RT-PCR.

RNA isolation from *A. baumannii*, cDNA generation, and quantitative reverse transcription-PCR (qRT-PCR) using SYBR green (Bio-Rad) were performed as previously described (46). The threshold cycle (*C_T_*) values for each transcript were normalized based on 16S rRNA levels.

### Statistical analyses.

All raw numerical data were saved in Excel files and imported into GraphPad Prism for statistical analysis. Specific statistical tests employed for each experiment are specified in the figure legends.

## SUPPLEMENTAL MATERIAL

Figure S1  (A) Growth over time in the presence of 125 µg/ml calprotectin. Wild-type and mutant strain Δ*mumT* harbor an empty plasmid, whereas Δ*mumT* p*mumT* expresses *mumT* from a constitutive promoter. (B) CFU recovered following 8 h of growth in the presence of 250 µg/ml calprotectin. (C) Calprotectin growth inhibition of mutant strains Δ*mumT* and Δ*mumC* is complemented by excess Mn. The percent growth of *A. baumannii* strains treated with 125 µg/ml calprotectin with or without the addition of 50 µM MnCl_2_ to the medium is shown. Growth relative to that in buffer alone is shown at 8 h. Experiments in panels B and C were combined from three independent experiments with three technical replicates each, and the means and standard errors of the means are graphed. Statistical significance for data in panels B and C was determined using a one-way analysis of variance with Dunnet’s multiple-comparisons test. (D and E) Growth of wild-type *A. baumannii* and strain Δ*mumT* in toxic concentrations of Fe (D) and Zn (E), graphed as the percentage of untreated cells at 8 h. Experimental results in panels D and E were combined from three independent experiments with three technical replicates each, and the mean and standard error of the mean of results were graphed. Significance for each condition (WT versus Δ*mumT* mutant) was determined using Student’s *t* test. (F) Zn and Fe levels of WT *A. baumannii* with an empty vector, strain Δ*mumT* with an empty vector, and strain Δ*mumT* with a vector containing *mumT* under control of the 16S promoter grown to mid-log phase. Cells were digested in metal-free nitric acid and analyzed by inductively coupled plasma mass spectrometry. Data were combined from three biological replicates that were measured in technical triplicate. The differences in mean values (WT versus strain Δ*mumT*) for each metal were not significant (Student’s *t* test). *, *P* < 0.05; **, *P* < 0.01; ***, *P* < 0.001. Download Figure S1, TIF file, 96.2 MB

Figure S2  (A) cDNA was amplified from RNA isolated from wild-type *A. baumannii* using reverse transcriptase (RT) and random primers. PCR was performed using this cDNA as the template and primer pairs designed to amplify across the junctions of predicted open reading frames. Primer annealing positions are shown schematically by arrows. cDNA samples prepared without RT were included as negative controls, and gene internal primer pairs were included as positive controls. (B) Wild-type *A. baumannii* and isogenic mutants Δ*mumT*, Δ*mumU*, Δ*mumH*, and Δ*mumC* were subcultured in LB 1:50 for 1 h and then inoculated 1:100 into LB for growth curve analysis. Optical density at 600 nm was monitored over time. (C) Growth of wild-type *A. baumannii* and strains Δ*mumT* and Δ*mumT* expressing *mumT* from a constitutive promoter in *trans* in 700 mM urea. Optical density was monitored over time. (D) Growth of *A. baumannii* and strain Δ*mumT* and strain Δ*mumT* p.*mumT* in 700 mM urea relative to growth in LB alone at 8 h. Data in panels B, C, and D were combined from three separate experiments with three technical replicates each and depict the mean and standard errors of the means of the results. Significance was calculated using a one-way analysis of variance with Dunnet’s multiple-comparisons test. *, *P* < 0.05; **, *P* < 0.01; ***, *P* < 0.001. Download Figure S2, TIF file, 83.1 MB

Figure S3  (A) Growth of wild-type *A. baumannii* and mutant strains Δ*mumL* and Δ*mumU* in the presence of 125 µg/ml calprotectin. (B) PCR products amplified (35 cycles) from 1:100 dilutions of cDNA synthesized from 2 µg of DNase-treated RNA. PCR products were amplified from primers specific to *mumT* or 16S*.* RNA was isolated from WT *A. baumannii* and mutant strains Δ*mumC* and Δ*mumT* grown in LB to early exponential phase. No reverse transcriptase samples were included as a negative control. (C and D) The growth deficit of strain Δ*mumC* in calprotectin is complemented by the expression of *mumC* on a plasmid under a constitutive promoter. (C) Growth was monitored by the optical density at 600 nm (OD_600_) over time. (D) Growth relative to buffer alone is shown at 24 h. For panels B and C, data were combined from three independent experiments with three technical replicates in each experiment and depict the means and standard errors of the means. Significance was calculated using a one-way analysis of variance with Dunnet’s multiple-comparisons test. (E) Growth of wild-type *A. baumannii* and strain Δ*mumC* in LB supplemented with toxic concentrations of MnCl_2_ (2.5 mM to 5 mM, as indicated on the graph). Growth relative to that of the untreated control is shown at 10 h. Data were combined from three independent experiments with three technical replicates in each experiment and depict the mean and standard error of the mean results. Significance was calculated using Student’s *t* test comparing two groups at each concentration of Mn. *, *P* < 0.05; **, *P* < 0.01. Download Figure S3, TIF file, 89.4 MB

Table S1  Bacterial strains used in this study. Table S1, DOCX file, 0.02 MB

Table S2  Primers used in this study. Table S2, DOCX file, 0.02 MB
