# Supplementary material for: Acinetobacter baumannii Coordinates Urea Metabolism with Metal Import To Resist Host-Mediated Metal Limitation
Source: mBio. 2016 Sep 27;7(5):e01475-16. doi: 10.1128/mBio.01475-16 (PMC5050338; doi:10.1128/mBio.01475-16)
Supplement: Table S1 — Bacterial strains used in this study. [file mbo005163003st1.docx]

**Supplemental Table 1: Bacterial strains used in this study.**

| **Strain** | **Relevant characteristics** | **Reference or source** |
| --- | --- | --- |
| 17978 | Wild-type | ATCC |
| Δ*znuB* | In-frame Δ*znuB::aphA* | ([13](#_ENREF_13)) |
| Δ*mumT* | In-frame Δ*mumT::aphA* | This study |
| Δ*mumR* | In-frame Δ*mumR::aphA* | This study |
| Δ*mumL* | In-frame Δ*mumL::aphA* | This study |
| Δ*mumU* | In-frame Δ*mumU::aphA* | This study |
| Δ*mumH* | In-frame Δ*mumH::aphA* | This study |
| Δ*mumC* | In-frame Δ*mumC::aphA* | This study |
| Δ*mumT* pWH1266 | Empty vector control | This study |
| 17978 pWH1266 | Empty vector control | ([13](#_ENREF_13)) |
| Δ*mumT* p.*mumT* | Complementation strain containing plasmid p.P*_r01_*.*mumT.*WH1266 | This study |
| Δ*mumC* pWH1266 | Empty vector control (p.P*_r01_*.WH1266) | This study |
| Δ*mumC* p.*mumC* | Complementation strain containing plasmid p.P*_r01_*.*mumC.*WH1266 | This study |
| 17978 p.P*_mumT_*.*lux* | Reporter strain containing plasmid p.P*_mumT_*.*luxABCDE.*MU368.*tet* | This study |
| Δ*mum*R p.P*_mumT_*.*lux* | Reporter strain containing plasmid p.P*_mumT_*.*luxABCDE.*MU368.*tet* | This study |
| *Escherichia coli* DH5α pFLP2 | Cloning strain containing *A. baumannii* suicide vector | ([54](#_ENREF_54)) |
| *Escherichia coli* DH5α p.*mumT::aphA*.FLP2 | Cloning strain containing *mumT* knockout vector | This study |
| *Escherichia coli* DH5α p.*mumR::aphA*.FLP2 | Cloning strain containing *mumR* knockout vector | This study |
| *Escherichia coli* DH5α p.*mumL::aphA*.FLP2 | Cloning strain containing *mumL* knockout vector | This study |
| *Escherichia coli* DH5α p.*mumU::aphA*.FLP2 | Cloning strain containing *mumU* knockout vector | This study |
| *Escherichia coli* DH5α p.*mumH::aphA*.FLP2 | Cloning strain containing *mumH* knockout vector | This study |
| *Escherichia coli* DH5α p.*mumC::aphA*.FLP2 | Cloning strain containing *mumC* knockout vector | This study |
| *Escherichia coli* DH5α p.P*_r01_*.WH1266 | Cloning strain containing plasmid p.P*_r01_*.WH1266 | (46) |
| *Escherichia coli* DH5α p.*mumT* | Cloning strain containing plasmid p.P*_r01_*.*mumT.*WH1266 | This study |
| *Escherichia coli* DH5α p.*mumC* | Cloning strain containing plasmid p.P*_r01_*.*mumC.*WH1266 | This study |
| *Escherichia coli* DH5α p.P*_mumT_*.*lux* | Cloning strain containing plasmid p.P*_mumT_*.*lux* | This study |
| *Escherichia coli* DH5α pMU368 | Cloning strain containing plasmid pMU368 | (47) |
| *Escherichia coli* DH5α pXen1 | Cloning strain containing plasmid pXen1 | (48) |
| *Escherichia coli* DH5α  p.*luxABCDE.*MU368 | Cloning strain containing plasmid p.*luxABCDE.*MU368 | This study |
| *Escherichia coli* DH5α  p.*luxABCDE.*MU368.*tet* | Cloning strain containing plasmid p.*luxABCDE.*MU368.*tet* | This study |
