# Supplementary material for: Acinetobacter baumannii Coordinates Urea Metabolism with Metal Import To Resist Host-Mediated Metal Limitation
Source: mBio. 2016 Sep 27;7(5):e01475-16. doi: 10.1128/mBio.01475-16 (PMC5050338; doi:10.1128/mBio.01475-16)
Supplement: Table S2 — Primers used in this study. [file mbo005163003st2.docx]

**Supplemental Table 2: Primers used in this study.**

| **Primer** | **Primer Sequence** | **Primer description** |
| --- | --- | --- |
| 1265_FL1F | ATA TAA GGA TCC GGC AGA AGA AAC AGA AAA ATA T | 5’ primer for amplifying upstream flanking region for *mumR* knockout construct |
| 1265_FL1R | CTAGTTAGTCACATATGGCCTTTATGGGTC | 3’ primer for amplifying upstream flanking region for *mumR* knockout construct |
| 1265_FL2F | GAGGGAATAATGACATATGGCTATTTCTTGAATG | 5’ primer for amplifying downstream flanking region for *mumR* knockout construct |
| 1265_FL2R | CAT TAT TCT AGA TCC GGC ATA ACA GAT ATA ACC AC | 3’ primer for amplifying downstream flanking region for *mumR* knockout construct |
| 1265_K1F | GACCCATAAAGGCCATATGTGACTAACTAG | 5’ primer for amplifying *aphA* for *mumR* knockout construct |
| 1265_K1R | CATTCAAGAAATAGCCATATGTCATTATTCCCTC | 3’ primer for amplifying *aphA* for *mumR* knockout construct |
| 1266_FL1F | atataaGGATCCCAACAGGAATTCCCAAAA | 5’ primer for amplifying upstream flanking region for *mumT* knockout construct |
| 1266_FL1R | CCTCCTAGTTAGTCACATATGAAAAATTGAGGC | 3’ primer for amplifying upstream flanking region for *mumT* knockout construct |
| 1266_FL2F | TGGAGGGAATAATGACATATGGGGTAATTATAACTA | 5’ primer for amplifying downstream flanking region for *mumT* knockout construct |
| 1266_FL2R | ctatgtTCTAGAAAACCTGCACGGATTTTTAAAC | 3’ primer for amplifying downstream flanking region for *mumT* knockout construct |
| 1266_K1F | GCCTCAATTTTTCATATGTGACTAACTAGGAGG | 5’ primer for amplifying *aphA* for *mumT* knockout construct |
| 1266_K1R | TAGTTATAATTACCCCATATGTCATTATTCCCTCCA | 3’ primer for amplifying *aphA* for *mumT* knockout construct |
| LEJ_54 | aagttcctattctctaggggGGCATGACTGTACATTTTG | 5’ primer for amplifying upstream flanking region for *mumC* knockout construct |
| LEJ_55 | tagttagtcaTTCATTCTCAAAATCTGTAAATTCG | 3’ primer for amplifying upstream flanking region for *mumC* knockout construct |
| LEJ_56 | tgagaatgaaTGACTAACTAGGAGGAATAAATG | 5’ primer for amplifying *aphA* for *mumC* knockout construct |
| LEJ_57 | gttcaaagctTCATTATTCCCTCCAGGTAC | 3’ primer for amplifying *aphA* for *mumC* knockout construct |
| LEJ_58 | ggaataatgaAGCTTTGAACTGTTTTAAATTG | 5’ primer for amplifying downstream flanking region for *mumC* knockout construct |
| LEJ_59 | ggttaaaaaggatcgatcctCGCAACTAAACCAAAGATC | 3’ primer for amplifying downstream flanking region for *mumC* knockout construct |
| LEJ_116 | AATATAGGATCCTACTGGCATTTCTGGTGATT | 5’ primer for amplifying upstream flanking region for *mumL* knockout construct |
| LEJ_117 | CCTCCTAGTTAGTCAGGCATTATCCCTTTATCAA | 3’ primer for amplifying upstream flanking region for *mumL* knockout construct |
| LEJ_118 | TTGATAAAGGGATAATGCCTGACTAACTAGGAGG | 5’ primer for amplifying *aphA* for *mumL* knockout construct |
| LEJ_119 | ATACATTGCGCTCTTCCTCATTATTCCCTC | 3’ primer for amplifying *aphA* for *mumL* knockout construct |
| LEJ_120 | GAG GGA ATA ATG AGG AAG AGC GCA ATG TAT | 5’ primer for amplifying downstream flanking region for *mumL* knockout construct |
| LEJ_121 | ataatatctagaAAAAAAACCAAAATTGTTTTTGCAGCG | 3’ primer for amplifying downstream flanking region for *mumL* knockout construct |
| LEJ_77 | aagttcctattctctaggggTTTAACGGATTGGTGTTG | 5’ primer for amplifying upstream flanking region for *mumU* knockout construct |
| LEJ_78 | tagttagtcaTGCGCTCTTCCTTATGCT | 3’ primer for amplifying upstream flanking region for *mumU* knockout construct |
| LEJ_79 | gaagagcgcaTGACTAACTAGGAGGAATAAATG | 5’ primer for amplifying *aphA* for *mumU* knockout construct |
| LEJ_80 | tttagctattTCATTATTCCCTCCAGGTAC | 3’ primer for amplifying *aphA* for *mumU* knockout construct |
| LEJ_81 | ggaataatgaAATAGCTAAAGAAGAATTAAAAAGG | 5’ primer for amplifying downstream flanking region for *mumU* knockout construct |
| LEJ_82 | ggttaaaaaggatcgatcctTTGATAGCTTGCAAAGTCC | 3’ primer for amplifying downstream flanking region for *mumU* knockout construct |
| LEJ_88 | aagttcctattctctaggggCAATTGATGGTGTATTTACCC | 5’ primer for amplifying upstream flanking region for *mumH* knockout construct |
| LEJ_89 | tagttagtcaTTGCTTATCCTTTTTAATTCTTC | 3’ primer for amplifying upstream flanking region for *mumH* knockout construct |
| LEJ_90 | ggataagcaaTGACTAACTAGGAGGAATAAATG | 5’ primer for amplifying *aphA* for *mumH* knockout construct |
| LEJ_91 | tctgtattcaTCATTATTCCCTCCAGGTAC | 3’ primer for amplifying *aphA* for *mumH* knockout construct |
| LEJ_92 | ggaataatgaTGAATACAGAAAAATTATTGATTG | 5’ primer for amplifying downstream flanking region for *mumH* knockout construct |
| LEJ_93 | ggttaaaaaggatcgatcctGACCTTGAGCTTTCGGTG | 3’ primer for amplifying downstream flanking region for *mumH* knockout construct |
| LEJ_140 | actcaGATATCatgatgaatacagaaaaattattgattg | 5’ primer for cloning *mumC* into complementation plasmid |
| LEJ_141 | aataaGGATCCttagtgaatactcgcaatagca | 3’ primer for cloning *mumC* into complementation plasmid |
| 1266_16S_pWH_F | atataaGGATCCGCTCAAATGGATGAGG | 5’ primer for cloning *mumT* into complementation plasmid |
| 1266_pWH_R | AGT GTG TCG ACT TAA AGC TTG GTC AAA TAA TTA | 3’ primer for cloning *mumT* into complementation plasmid |
| 1266_pro_Sac1 | ataattGAGCTCAAAGCTGTTTTGTAGGTAAAAC | 5’ primer for cloning *mumT* promoter into luminescence reporter construct |
| 1266_pro_BamH1 | aattctGGATCCTTAACAACGCTATTTTCAAATTG | 3’ primer for cloning *mumT* promoter into luminescence reporter construct |
| lux368For | TTA GGA TCC TGC AGA TGA AGC AAG AGG | 5’ primer for cloning *luxABCDE* from pXen1 into luminescence reporter construct |
| lux368Rev | GGC ACT AGT GTC GAC TCA ACT ATC AAA | 3’ primer for cloning *luxABCDE* from pXen1 into luminescence reporter construct |
| tetF | ATT AGC GGT ACC CGC TCG AAC AAG AGG ATG | 5’ primer for cloning *tet* cassette into luminescence reporter construct |
| tetR | TAA ATA GGT ACC GCG CCG TCC CGA TAA GAG A | 3’ primer for cloning *tet* cassette into luminescence reporter construct |
| 1266f | GGC CTA GAC ACA AAG TGG GG | *mumT* 5’ qRT-PCR primer |
| 1266r | AGC AAT CCC AAC ACG ATC ATG C | *mumT* 3’ qRT-PCR primer |
| r01RTf | CTGTAGCGGGTCTGAGAGGAT | *r01* 5’ qRT-PCR primer |
| r01RTr | CCATAAGGCCTTCTTCACAC | *r01* 3’ qRT-PCR primer |
| LEJ_23 | GAACCGGCAGGGCGTATTTC | *mumU* 5’ qRT-PCR primer |
| LEJ_24 | TACACTTGGCATACGCGCTG | *mumU* 3’ qRT-PCR primer |
| LEJ_27 | AGTGGCAGGCTCAAAAGTGC | *mumL* 5’ qRT-PCR primer |
| LEJ_28 | GCATCAATTACGGCTGCTGC | *mumL* 3’ qRT-PCR primer |
| LEJ_29 | AAAGACAGTCCGGGTGGTTG | *mumH* 5’ qRT-PCR primer |
| LEJ_30 | CCCGGTAACAGCAATGCAGG | *mumH* 3’ qRT-PCR primer |
| LEJ_31 | TAAAGCCGCGTTTGGTGGTG | *mumC* 5’ qRT-PCR primer |
| LEJ_32 | TGCTTCACGTACTGCCGACT | *mumC* 3’ qRT-PCR primer |
| LEJ_25 | GAAGGGGTACGTACAGGCGA | 5’ *mumR* primer for Supplemental Fig 2A |
| LEJ_34 | GCAATCAGCGCCGACACAAA | 3’ *A1S_1271* primer for Supplemental Fig 2A |
